# Hydrogen-bonded organic framework@conductive metal-organic framework heterostructures for ampere-level hydrogen peroxide production

**DOI:** 10.1038/s41467-025-65887-6

**Published:** 2025-12-04

**Authors:** Yingying Zou, Yulin Zhang, Chaoqi Zhang, Tong Bao, Yamin Xi, Niqi Ao, Zhijie Li, Yunying Wang, Chao Liu, Chengzhong Yu

**Affiliations:** 1 School of Chemistry and Molecular Engineering, East China Normal University, Shanghai, China; 2 State Key Laboratory of Petroleum Molecular and Process Engineering, SKLPMPE, East China Normal University, Shanghai, China; 3 Shanghai Frontiers Science Center of Molecule Intelligent Syntheses, School of Chemistry and Molecular Engineering, East China Normal University, Shanghai, China; 4 Australian Institute for Bioengineering and Nanotechnology, The University of Queensland, Brisbane, Queensland Australia

**Keywords:** Electrocatalysis, Sustainability, Metal-organic frameworks

## Abstract

Electrochemical two-electron oxygen reduction reaction (2e^–^ ORR) in neutral environments holds remarkable promise for sustainable hydrogen peroxide (H_2_O_2_) production. However, its practical application is largely hindered due to the scarcity of electrocatalysts with high selectivity and durability under ampere-level current densities. Herein, a hydrogen-bonded organic framework@conductive metal-organic framework (HOF@*c*MOF) heterostructure is designed for industrial-level H_2_O_2_ electrosynthesis. Through integrating DAT-HOF (DAT=diaminotriazole) and Co-*c*MOF, Co-N bonds formed at the heterointerface modulates the electronic structure of Co sites, optimizing the adsorption strength of oxygen intermediates with improved activity and selectivity. Besides, the formation of built-in electric field drives the proton migration from DAT-HOF to Co-*c*MOF, facilitating the O_2_ protonation to H_2_O_2_ at Co sites. In further combination with the high proton donation capability of DAT-HOF and high conductivity of Co-*c*MOF, efficient H_2_O_2_ production is achieved with a H_2_O_2_ Faradic efficiency of 97.1 ± 0.4%, a H_2_O_2_ yield of 738.9 mg h⁻^1^ cm⁻^2^ and a long-term durability over 100 h at 1200 mA cm⁻^2^. This work offers a high-performance electrocatalyst for promoting the industrial implementation of H_2_O_2_ electrosynthesis.

## Abstract (web summary)

Industrial-level electrochemical two-electron oxygen reduction reaction is promising for H_2_O_2_ production but lacks efficient catalysts. Here, the authors design a heterostructure catalyst for efficient H_2_O_2_ electrosynthesis at ampere-level current densities.

## Introduction

Hydrogen peroxide (H_2_O_2_) is a vital chemical extensively used in pharmaceuticals, paper manufacturing, chemical synthesis and environmental protection^1,2^. As a promising alternative to the traditional anthraquinone method, the electrochemical two-electron oxygen reduction reaction (2e^–^ ORR) process offers a sustainable and decentralized approach for H_2_O_2_ production across a wide pH range from 0–14^3–5^. However, the widely investigated reaction systems in acidic and alkaline mediums have limitations, including the H_2_O_2_ instability, corrosiveness and potential environmental issues^6–8^. In contrast, 2e^–^ ORR operated in mild neutral condition features environmental friendliness, low corrosion, high stability and flexible utilization of produced H_2_O_2_^9–11^. From a practical perspective, achieving neutral-condition H_2_O_2_ production via 2e^–^ ORR at ampere-level current densities (>300 mA cm^−2^)^12–14^ is of great significance. To date, various electrocatalysts such as noble metals^15^, carbons^16^, transition metal compounds^17^, single-atom materials^18,19^ and metal-organic frameworks (MOFs)^20–22^ have been applied for this purpose. However, most of them demonstrate high Faradaic efficiency (FE) only at modest current densities (<100 mA cm^−2^). The development of electrocatalysts that are capable of efficiently driving 2e^–^ ORR at high current densities up to ampere levels remains a challenge.

Among currently studied 2e^–^ ORR electrocatalysts, MOFs with large specific surface area, high porosity and tunable structures have garnered particular interest^23,24^. Compared to conventional MOFs, conductive MOFs (*c*MOFs) with π-conjugated frameworks exhibit high electrical conductivity and are ideal candidates for the design of advanced 2e^–^ ORR electrocatalysts^25–27^. However, the reported *c*MOF-based 2e^–^ ORR electrocatalysts (e.g., Mg-HITP^28^, ZnCu-MOF (H)^29^, Ni-HAB^30^) suffer from unsatisfactory performances in neutral electrolytes even at relatively low current densities, thus their application at industrial-relevant ampere-level current densities is rarely reported. The 2e^–^ ORR pathway involves a two-proton coupled two-electron transfer process (O_2_  +  H^+^  +  e^−^ → *OOH, *OOH  + H^+^ + e^−^ → H_2_O_2_) and competes with the 4e^–^ ORR pathway to generate H_2_O^31,32^. The precise modulation of catalytic active sites to suppress the 4e^−^ ORR pathway is essential for the selective production of H_2_O_2_. In addition to improving the selectivity of 2e^–^ ORR against 4e^–^ ORR, the proton supply is a critical factor that determines the formation of H_2_O_2_ in neutral environments^33,34^. Insufficient proton supply is detrimental to the generation of *OOH and the conversion of *OOH to H_2_O_2_, reducing the 2e^–^ ORR activity and selectivity^35^. At ampere-level current densities, the faster proton consumption imposes even higher demand on the proton supply^33^. To achieve ampere-level H_2_O_2_ electrosynthesis, the rational design of *c*MOF-based electrocatalysts with both modulated active sites and sufficient proton supply capability is an essential but challenging task.

As another important class of porous crystalline materials, hydrogen-bonded organic frameworks (HOFs) are formed by intermolecular hydrogen bonding (H-bond) of organic building blocks^36–38^. The versatile surface functional groups of HOFs enable effective hybridization with other materials, yielding heterostructures with synergistic properties^39^. Moreover, the chemical interaction at the heterointerface can induce charge redistribution and modulate the electronic structure of active sites, thereby enhancing the intrinsic activity and selectivity^40^. Importantly, the abundant hydrogen-bond networks in HOFs can facilitate proton conduction, potentially enhancing local proton donation during electrocatalysis^41^. Thus, we hypothesize that the construction of *c*MOFs/HOFs heterostructures is a promising approach to develop high-performance 2e^–^ ORR electrocatalysts. Such a design has been rarely reported.

**Fig. 1:**
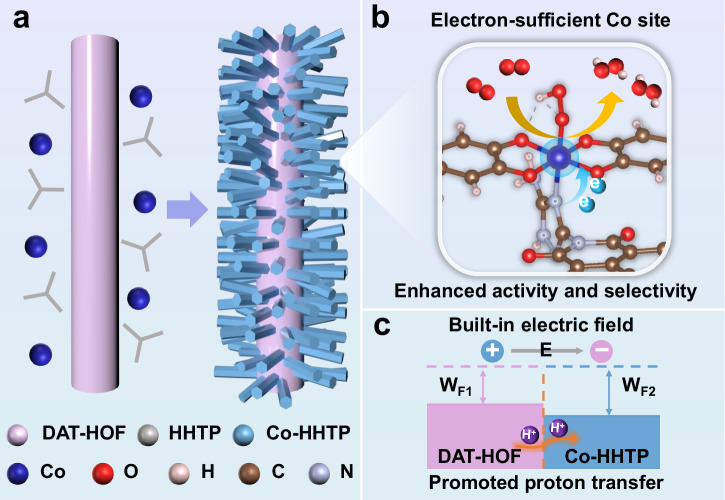
Synthesis and working mechanism of DAT-HOF@Co-HHTP. Schematic illustration of the **a** synthesis process, **b** interfacial active site and **c** built-in electric field of DAT-HOF@Co-HHTP heterostructure.

Herein, a HOF@*c*MOF heterostructure is constructed for ampere-level H_2_O_2_ electrosynthesis, exhibiting a H_2_O_2_ FE of 97.1 ± 0.4% and a H_2_O_2_ production rate of 738.9 mg h⁻^1^ cm⁻^2^ at 1200 mA cm^−2^, along with a long-term stability over 100 h. The conductive Co-HHTP (HHTP = 2,3,6,7,10,11-hexahydroxytripehenylene) is grown on DAT-HOF (DAT=diaminotriazole) with sufficient proton donation ability, resulting in a unique rod-on-rod heterostructure (Fig. 1a). At the HOF-*c*MOF heterointerface, the formation of Co-N bonds results in the electron-enriched Co active sites with optimized adsorption strength of oxygen intermediates, making pivotal contributions to the enhanced 2e^–^ ORR activity and selectivity (Fig. 1b). Furthermore, the charge redistribution between two components with different Fermi levels induces the formation of a built-in electric field, driving the directional migration of protons from DAT-HOF to Co-HHTP for facilitating the protonation of O_2_ to H_2_O_2_ at Co sites (Fig. 1c). By integrating modulated active sites, high electron conductivity and enhanced hydrogenation capability, the rationally designed heterostructure delivers excellent 2e^–^ ORR performance at ampere-level current densities, superior than most reported electrocatalysts. Our work paves the way for the design of advanced electrocatalysts for practical H_2_O_2_ production.

## Results

To prepare the DAT-HOF@Co-HHTP heterostructure, DAT-HOF was firstly synthesized through the imidization reaction according to a reported protocol^42,43^. Scanning electron microscope (SEM) and transmission electron microscopy (TEM) images of DAT-HOF (Supplementary Fig. 1a, b) show a uniform one-dimensional (1D) rod-like morphology with micrometer-scale length and smooth surface. The crystalline structure of DAT-HOF was studied by powder X-ray diffraction (PXRD). The experimental PXRD pattern (Supplementary Fig. 1c) matches well with the simulated profile via Pawley refinement (R_p_ = 5.26% and R_wp_ = 6.76%), revealing a hexagonal crystal structure with cell parameters of *a* = 14.25, *b* = 14.25 Å, *c* = 5.17 Å.

**Fig. 2:**
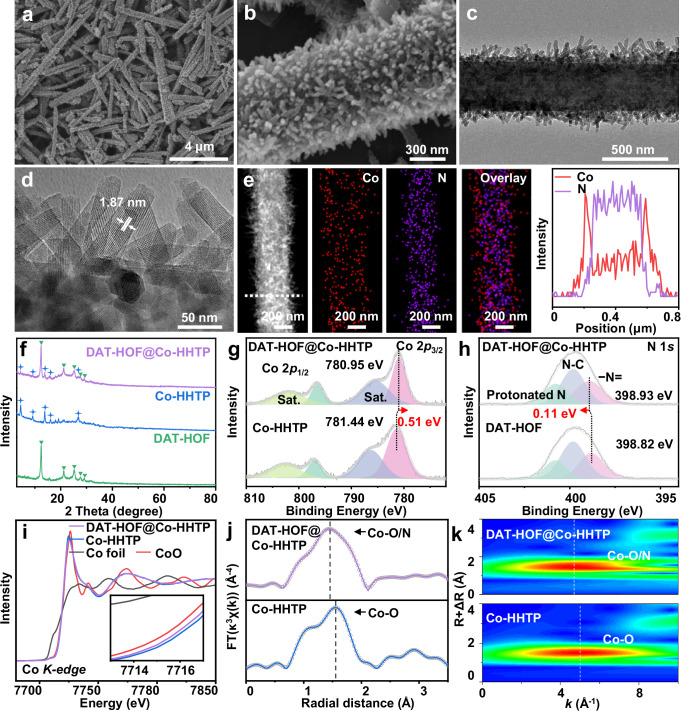
Morphology and structural characterization. **a**, **b** SEM, **c** TEM, **d** HRTEM, and **e** HADDF-STEM and corresponding EDS mapping images of DAT-HOF@Co-HHTP. Inset in **e** is the line scanning spectra. **f** XRD patterns of DAT-HOF@Co-HHTP, DAT-HOF and Co-HHTP. **g** Co 2*p* spectra of DAT-HOF@Co-HHTP and Co-HHTP and **h** N 1 *s* spectra of DAT-HOF@Co-HHTP and DAT-HOF. **i** Co K-edge of XANES spectra, **j** Co K-edge FT-EXAFS spectra, and **k** wavelet transforms for Co K-edge EXAFS of DAT-HOF@Co-HHTP and Co-HHTP. Source data for Fig. 2 are provided as a Source Data file.

The subsequent reaction of DAT-HOF with cobalt (II) acetate and HHTP ligands resulted in the DAT-HOF@Co-HHTP heterostructure. The low-magnification SEM image of DAT-HOF@Co-HHTP (Fig. 2a) shows a well-preserved microrod-like morphology with rough surface. At higher magnification (Fig. 2b), high-density nanorods with an average diameter of ~30 nm as the shell adhered to the microrod as the core are observed. The core-shell structured rod-on-rod heterostructure is further demonstrated by TEM studies (Fig. 2c). In the high-resolution TEM (HRTEM) image of the shell nanorods (Fig. 2d), the distinct lattice fringes with an interplanar distance of 1.87 nm are ascribed to the (100) planes of Co-HHTP. Figure 2e displays the high-angle annular dark-field scanning TEM (HAADF-STEM) and energy-dispersive X-ray spectroscopy (EDX) element mapping images of DAT-HOF@Co-HHTP. The Co and N elements predominately exist in the internal core and external shell regions, respectively. The line scanning spectra collected along the short axis (inset in Fig. 2e) further indicate the N-rich DAT-HOF core and Co-rich Co-HHTP shell in the rod-on-rod heterostructure.

To analyze the crystalline structure and chemical composition of DAT-HOF@Co-HHTP, XRD and Fourier transform infrared (FTIR) spectroscopy measurements were conducted. In addition to DAT-HOF, Co-HHTP nanorods were also prepared as a control (Supplementary Fig. 2). As shown in Fig. 2f, two groups of diffraction peaks attributed to DAT-HOF and Co-HHTP are simultaneously observed in the XRD pattern of DAT-HOF@Co-HHTP. In the FTIR spectrum of DAT-HOF (Supplementary Fig. 3a), the peaks located at 1410 and 1060 cm^−1^ are attributed to the axial C=N−C stretching vibration of imine and in-plane rocking vibration of −NH_2_^43^. Besides, the peak at 1657 cm^−1^ corresponds to the stretching mode of C=NH^+^ bond, originated from the protonation of C=N group in DAT-HOF (Supplementary Fig. 3b)^44^. Compared to DAT and NTD ligands, the additional peaks at 3247 and 3066 cm^−1^ in the spectrum of DAT-HOF are associated with the stretching vibration of H-bond N–H (Supplementary Fig. 4), indicating the formation of hydrogen bonding in DAT-HOF^45,46^. For Co-HHTP, the peaks at 1454, 1298 and 1216 cm^−1^ in the spectrum are assigned to the benzene skeleton vibrations of HHTP^47^. By integrating DAT-HOF and Co-HHTP, the typical peaks of two components are simultaneously detected in DAT-HOF@Co-HHTP. Supplementary Fig. 5 shows the N_2_ adsorption-desorption isotherms of the samples. The Brunauer-Emmett-Teller (BET) specific surface areas and pore volumes of DAT-HOF, Co-HHTP and DAT-HOF@Co-HHTP were determined to be 75.1 m^2^ g^−1^ and 0.29 cm^3 ^g^−1^ for DAT-HOF, 91.9 m^2^ g^−1^ and 0.46 cm^3 ^g^−1^ for DAT-HOF@Co-HHTP, and 118.3 m^2^ g^−1^ and 0.50 cm^3 ^g^−1^ for Co-HHTP, respectively.

X-ray photoelectron spectroscopy (XPS) study was employed to investigate the surface chemical states of the three samples. The survey spectra (Supplementary Fig. 6) show the co-existence of Co, O, N and C elements in DAT-HOF@Co-HHTP heterostructure. The Co 2*p* spectrum of Co-HHTP (Fig. 2g) was deconvoluted into Co 2*p*_1/2_ and Co 2*p*_3/2_ orbitals of Co^2+^ at 797.04 and 781.44 eV, respectively, with two satellite peaks at 802.62 and 786.38 eV. Compared to Co-HHTP, the binding energy of Co 2*p*_3/2_ orbital of Co^2+^ in DAT-HOF@Co-HHTP decreases by 0.51 eV. In the N 1 *s* spectrum of DAT-HOF (Fig. 2h), three main peaks are observed at 398.82, 399.81 and 400.80 eV, corresponding to −N=, N−C and protonated N^44^, respectively, consistent with the FTIR observations. After hybridization with Co-HHTP, the peak positions of N−C and protonated N are almost unchanged, while the binding energy of −N= peak is elevated by ≈0.11 eV. The opposite change trend of N 1 *s* and Co 2*p* indicates the electron transfer from DAT-HOF to Co-HHTP via the Co−N bonds that are formed by the interaction between Co^2+^ and −N=.

X-ray absorption spectroscopy (XAS) measurements were carried out to further elucidate the electronic structure of DAT-HOF@Co-HHTP, with Co-HHTP as comparison. As shown in the X-ray absorption near-edge structure (XANES) spectra (Fig. 2i), the energy position of Co K-edge for DAT-HOF@Co-HHTP is more negative than Co-HHTP, indicating the formation of electron-enriched Co sites in the heterostructure via electron transfer from DAT-HOF to Co-HHTP. Figure 2j presents the Fourier transformed-extended X-ray absorption fine structure (FT-EXAFS) spectra of DAT-HOF@Co-HHTP and Co-HHTP. In the Co K-edge spectrum of Co-HHTP, the main peak at ≈1.58 Å is assigned to the Co−O bond, which originates from the coordination of Co^2+^ with HHTP ligands and H_2_O. For DAT-HOF@Co-HHTP, the peak exhibits a slight negative shift of 0.32 Å compared to Co-HHTP, possibly resulting from the formation of Co–N bond at DAT-HOF/Co-HHTP interface. The hypothesis is further supported by the wavelet transform profiles derived from the EXAFS spectra (Fig. 2k). The XAS observations are well consistent with XPS results, collaboratively verifying the successful construction of chemically bonded DAT-HOF@Co-HHTP heterostructure.

**Fig. 3:**
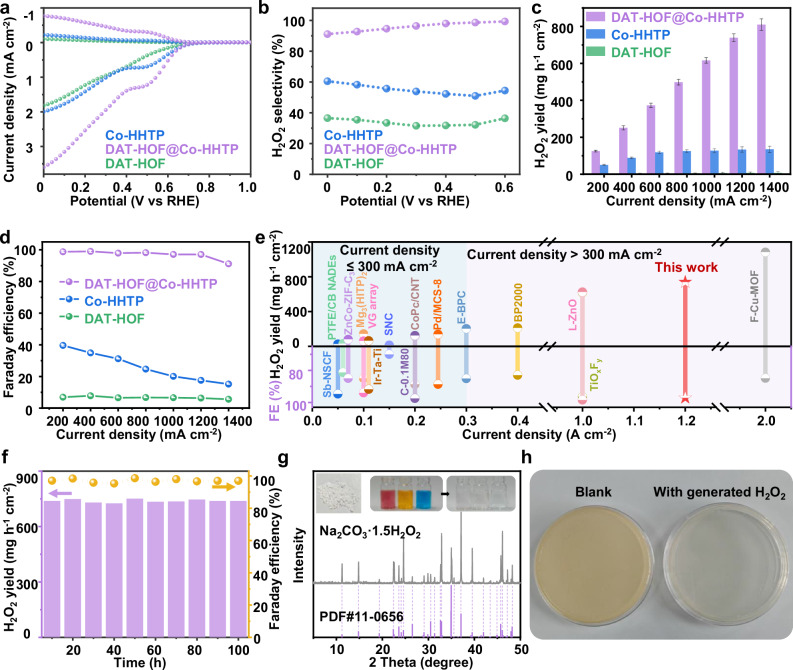
Electrocatalytic 2e⁻ ORR performance. **a** LSV polarization curves, **b** H_2_O_2_ selectivity, **c** H_2_O_2_ production rate and **d** Faraday efficiency of DAT-HOF@Co-HHTP, DAT-HOF and Co-HHTP. **e** Performance comparison with reported electrocatalysts. **f** H_2_O_2_ FE and production rate versus time at a current density of 1200 mA cm^–2^. **g** XRD pattern of extracted solid H_2_O_2_ (insets are photographs of solid H_2_O_2_ and degradation of different dyes by solid H_2_O_2_). **h** In situ generated H_2_O_2_ for disinfection of Staphylococcus aureus. Error bars represent the standard error of the mean from three independent experimental measurements. All the curves were used without IR compensation. Source data for Fig. 3 are provided as a Source Data file.

The ORR catalytic performance of DAT-HOF@Co-HHTP heterostructure for H_2_O_2_ production was evaluated using a three-electrode cell in an O_2_-saturated 0.1 M phosphate buffer saline (PBS) solution (pH = 7). Linear sweep voltammetry (LSV) measurement was first performed using a rotating ring-disk electrode (RRDE) system at 1600 rpm. The ORR currents were recorded at the disk electrode (solid line) and the oxidation of generated H_2_O_2_ was detected via the Pt ring electrode (dashed line). A shown in the LSV curves (Fig. 3a), DAT-HOF@Co-HHTP exhibits the most positive onset potential (the potential at 0.10 mA cm^−2^) of 0.67 V vs. RHE among all the samples, indicative of the highest ORR activity. Besides, the Tafel slope of DAT-HOF@Co-HHTP was determined to be 71.3 mV dec⁻^1^, lower than that of Co-HHTP (89.4 mV dec⁻^1^) and DAT-HOF (132.8 mV dec⁻^1^, Supplementary Fig. 7a), suggesting the fastest reaction kinetics of DAT-HOF@Co-HHTP. The 2e⁻ ORR selectivity was calculated based on the disk and ring currents, then plotted as a function of the applied potential in Fig. 3b. The 2e⁻ ORR selectivity of DAT-HOF@Co-HHTP exceeds 90.0% across a wide potential range from 0.0 to 0.6 V vs. RHE with a peak value of 99.3% at 0.6 V vs. RHE, superior than Co-HHTP (54.4% at 0.6 V vs. RHE) and DAT-HOF (36.4% at 0.6 V vs. RHE). The electron transfer numbers (n) of DAT-HOF, Co-HHTP and DAT-HOF@Co-HHTP were calculated to be 3.32, 2.89 and 2.08, respectively (Supplementary Fig. 7b), manifesting the high 2e^−^ ORR selectivity of DAT-HOF@Co-HHTP.

Moreover, the LSV polarization curves measured in the flow electrolytic cell system (Supplementary Figs. 8 and 9) show that DAT-HOF@Co-HHTP exhibits increased ORR current density and activity than DAT-HOF and Co-HHTP, consistent with the results obtained from the RRDE measurements. The H_2_O_2_ production rate and Faraday efficiency (FE) were further calculated as displayed in Fig. 3c, d, respectively. DAT-HOF@Co-HHTP exhibits increased H_2_O_2_ production rates from 125.3 mg h⁻^1^ cm⁻^2^ at 200 mA cm^−2^ to 738.9 mg h⁻^1^ cm⁻^2^ at 1200 mA cm^−2^, all with a high H_2_O_2_ FE (97.1 ± 0.4% to 99.0 ± 0.6%). At a higher current density of 1400 mA cm^−2^, even with a slight drop of 5.9% for H_2_O_2_ FE (still exceeding 90%), a H_2_O_2_ generation rate of 809.6 mg h⁻^1^ cm⁻^2^ is achieved, suggesting the efficient conversion of O_2_ to H_2_O_2_ at ampere level. For Co-HHTP, the H_2_O_2_ production rate is elevated at relatively low current densities from 200 to 600 mA cm^−2^, but quickly reaches a plateau after 600 mA cm^−2^ with a peak value of 134.9 mg h⁻^1^ cm⁻^2^. In contrast to the well-maintained and high H_2_O_2_ FE values of DAT-HOF@Co-HHTP, the H_2_O_2_ FE of Co-HHTP is dramatically decreased from 39.6 ± 0.4% to 15.2 ± 0.1%. For DAT-HOF, the overall H_2_O_2_ production rate remains limited (~1.7 to 7.7 mg h⁻^1^ cm⁻^2^).

Notably, the performance of DAT-HOF@Co-HHTP especially at ampere level is superior to most reported 2e⁻ ORR electrocatalysts including noble metal nanoparticles, transition metal compounds, metal-free carbon materials and other MOFs (Fig. 3e and Supplementary Table 1). Moreover, even after 100 h of continuous electrolysis, the H_2_O_2_ FE and production rate of DAT-HOF@Co-HHTP decreased by only 1.6% and 11.6 mg h⁻^1^ cm⁻^2^, respectively (Fig. 3f). Additionally, the chronoamperometry (I-t) curve shows a nearly constant current density during a long-period operation of 100 h (Supplementary Fig. 10), further demonstrating the good electrochemical stability of DAT-HOF@Co-HHTP. Additionally, DAT-HOF@Co-HHTP after test was collected and characterized by SEM, TEM, XRD and XPS (Supplementary Fig. 11). The results show that the rod-on-rod morphology, crystal and electronic structure are well preserved, demonstrating the long-term durability.

By further adding Na_2_CO_3_ into the electrolyte after a successive electrolysis of 100 h, the produced H_2_O_2_ can be extracted in the form of sodium carbonate perhydrate (Na_2_CO_3_·1.5H_2_O_2_) as a solid H_2_O_2_ product. The XRD pattern of collected powder shows well-matched diffraction peaks with those of standard pattern, verifying the formation of solid H_2_O_2_ (Fig. 3g)^48^. The variation in relative peak intensity may be attributed to the difference in preferred orientation between solid and standard sample. Additionally, the Raman spectra of solid H_2_O_2_ and standard Na_2_CO_3_·1.5H_2_O_2_ (Supplementary Fig. 12) show almost identical patterns, further verifying the successful preparation of solid H_2_O_2_. When directly used as an oxidant, the as-synthesized solid H_2_O_2_ powder exhibits high oxidative activity for efficient degradation of various organic dyes (Fig. 3g). The preparation of solid H_2_O_2_ offers a facile approach for reducing the storage and transportation costs of liquid H_2_O_2_^49^. Moreover, the generated H_2_O_2_ can inhibit the growth of Staphylococcus aureus, indicating its superior antibacterial application potential (Fig. 3h).

To further evaluate the electrochemical property, the measurements of electrochemical surface area (ECSA) and electrochemical impedance spectroscopy (EIS) were conducted. As determined by the double-layer capacitances (C_dl_) in the non-Faradic region (1.03–1.13 V vs. RHE, Supplementary Fig. 13), the ECSA values of the samples show the trend of DAT-HOF@Co-HHTP (0.30 mF cm⁻^2^) > Co-HHTP (0.18 mF cm⁻^2^) > DAT-HOF (0.08 mF cm⁻^2^), indicating the higher intrinsic activity of DAT-HOF@Co-HHTP^20^. The EIS spectra (Supplementary Fig. 14) show that the charge-transfer resistance (R_ct_) of DAT-HOF@Co-HHTP (25.4 Ω cm⁻^2^) is slightly larger than that of Co-HHTP (20.6 Ω cm⁻^2^), but significantly lower than DAT-HOF (59.7 Ω cm⁻^2^), suggesting the enhanced charge transportation capability of DAT-HOF@Co-HHTP than DAT-HOF by the integration with conductive Co-HHTP.

**Fig. 4:**
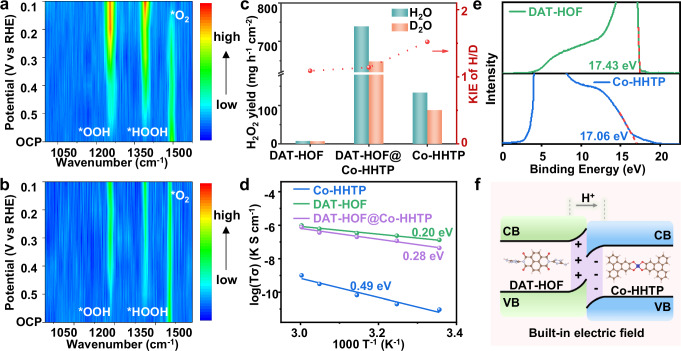
Mechanistic studies. Contour maps derived from the in situ ATR-IR spectra recorded on **a** DAT-HOF@Co-HHTP and **b** Co-HHTP at different applied potentials. The color bars represent intensity with arb. units. **c** Arrhenius plots at different temperatures for DAT-HOF, Co-HHTP and DAT-HOF@Co-HHTP. **d** Kinetic isotope effect (KIE) of H_2_O/D_2_O for ORR on DAT-HOF, Co-HHTP and DAT-HOF@Co-HHTP. **e** UPS spectra of DAT-HOF and Co-HHTP. **f** Built-in electric field in DAT-HOF@Co-HHTP. Source data for Fig. 4 are provided as a Source Data file.

In situ attenuated total reflection infrared spectroscopy (ATR-IR) was applied to monitor the formation of adsorbed oxygen intermediates on DAT-HOF@Co-HHTP and Co-HHTP during the ORR process. As shown in the ATR-IR spectrum of DAT-HOF@Co-HHTP at open circuit potential (OCP, Fig. 4a and Supplementary Fig. 15a), the peak at 1488 cm⁻^1^ is attributed to the O–O stretching mode of adsorbed molecular oxygen (*O_2_)^50^. With the applied potential decreased to 0.5 V vs. RHE, the peak intensity of *O_2_ diminishes with the generation of two new peaks of *OOH and *HOOH at 1255 and 1396 cm⁻^1^, respectively, indicating the conversion of O_2_ to oxygen intermediates^50^. By further lowering the potential from 0.5 to 0.1 V vs. RHE, *O_2_ is dramatically consumed with the accumulation of *OOH and *HOOH. Compared to DAT-HOF@Co-HHTP, the peak intensity of *O_2_ at OCP for Co-HHTP is obviously weaker, revealing the enhanced O_2_ adsorption on DAT-HOF@Co-HHTP (Fig. 4b and Supplementary Fig. 15b). At more negative potentials, the conversion of *O_2_ to *OOH and *HOOH is significantly retarded, suggesting the promoted 2e^–^ ORR process over DAT-HOF@Co-HHTP, in accordance with the electrocatalytic results.

To understand the enhanced performance of DAT-HOF@Co-HHTP, the proton supply and transfer properties that greatly affects the hydrogenation step during 2e⁻ ORR are investigated. Firstly, the H_2_O/D_2_O kinetic isotope effect (KIE) experiment was conducted to explore the role of proton supply in O_2_ hydrogenation to H_2_O_2_^51^. Generally, the KIE value (the ratio of H_2_O_2_ yield in H_2_O and D_2_O) closer to 1 indicates that the proton donation is not the rate-determining step (RDS). As shown in Fig. 4c, the KIE value of Co-HHTP is measured to be 1.52, indicating the inferior proton supply capability. After integrating DAF-HOF (KIE value of 1.09), the KIE value of DAT-HOF@Co-HHTP decreases to 1.14, showing enhanced proton supply for H_2_O_2_ production.

Subsequently, the proton conductivity (σ) of the samples was determined by alternating current impedance test under 95% relative humidity from 273 to 333 K^52^. As displayed in Supplementary Fig. 16, the σ value of DAT-HOF@Co-HHTP increases with temperature and reaches 6.64 × 10^–4^ S cm^–1^ at 333 K. The value is slightly lower than that of pristine DAT-HOF (7.35 × 10^–4^ S cm^–1^), which can be attributed to the integration of Co-HHTP with a relatively lower proton conductivity (σ of 3.73 × 10^–6^ S cm^−^^1^). Based on the corresponding Arrhenius plots, the activation energies (E_a_) in proton conduction are calculated^53^. According to literature, the proton transportation on solid materials follows two mechanisms: (1) the Grotthuss mechanism involves the proton diffusion via a H-bond network with E_a_ between 0.1-0.4 eV^54^; (2) the vehicle mechanism describes the migration of protons coupled with movable carriers (e.g., H_2_O) with E_a_ > 0.4 eV^55^. As shown in Fig. 4d, the E_a_ value of Co-HHTP is calculated to be 0.49 eV, corresponding to a vehicular mechanism. In contrast, the proton transfer on DAT-HOF@Co-HHTP proceeds via the Grotthuss mechanism with a lower E_a_ of 0.28 eV, which is mainly contributed by the core composition (DAT-HOF) with a H-bond network (E_a_ of 0.2 eV)^56^.

To elucidate the proton transfer at the DAT-HOF/Co-HHTP heterointerface, ultraviolet photoelectron spectroscopy (UPS) analysis was performed for determining the work function (Φ) of DAT-HOF and Co-HHTP using a monochromatic He light source (21.22 eV). The secondary electron cutoff energies (E_cutoff_) of DAT-HOF and Co-HHTP are 17.43 and 17.06 eV (Fig. 4e), respectively. Based on the equation of Φ = 21.22 – E_cutoff_, the Φ values of DAT-HOF and Co-HHTP were determined to be 3.79 and 4.16 eV (vs vacuum), respectively^57^. According to the formula of E_f_ = E_v_ – Φ (E_v_ is the vacuum level, assumed as 0 eV), the Fermi levels (E_f_) of DAT-HOF and Co-HHTP are calculated as –3.79  and –4.16 eV (vs vacuum). When DAT-HOF and Co-HHTP are in contact at the heterostructure, the difference in E_f_ induces the spontaneous migration of free electrons from DAT-HOF to Co-HHTP until the E_f_ equilibrium is established^58^. A built-in electric field directed from DAT-HOF to Co-HHTP is thus formed (Fig. 4f), offering driving force for promoting the proton transportation from DAT-HOF to Co-HHTP. Based on the results of in-situ ATR-IR, KIE, proton conductivity and UPS measurements, it is demonstrated that DAT-HOF with protonated structure serves as an efficient proton source, while the abundant hydrogen-bond networks provide highways for proton transfer with low energy barrier. Further contributed by the built-in electric field, DAT-HOF@Co-HHTP exhibits sufficient proton supply capability for O_2_ hydrogenation toward H_2_O_2_ production.

**Fig. 5:**
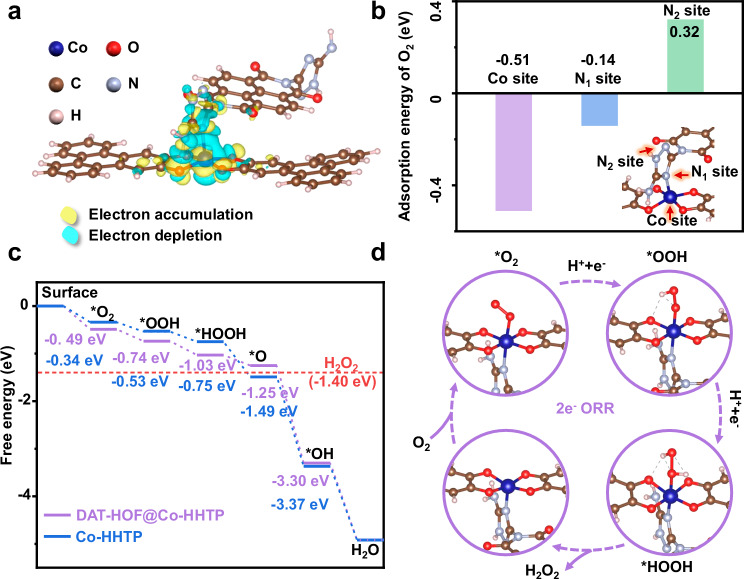
DFT calculations. **a** Calculated charge density difference of DAT-HOF@Co-HHTP heterostructure. **b** Calculated O_2_ adsorption energies at the Co, N_1_, and N_2_ site of DAT-HOF@Co-HHTP, respectively. **c** Free energy diagrams for ORR over Co-HHTP and DAT-HOF@Co-HHTP heterostructure. **d** Schematic diagram of the ORR pathways over DAT-HOF@Co-HHTP heterostructure. Source data for Fig. 5 are provided as a Source Data file.

Furthermore, density functional theory (DFT) simulation was conducted to gain deeper insights into the 2e⁻ ORR process over DAT-HOF@Co-HHTP. The XPS observations suggest the interaction between DAT-HOF and Co-HHTP through cobalt-imine nitrogen coordination. Considering that there are two types of imine nitrogen in DAT-HOF including N_1_ and N_2_ as indicated in Supplementary Fig. 17a, the formation energies of two possible heterostructure models are firstly calculated (Supplementary Fig. 17b, c). The results (Supplementary Fig. 17 d) show that the formation energy of the heterostructure via Co-N_1_ interaction ( − 0.96 eV) is more negative than the Co-N_2_ counterpart ( − 0.79 eV). The DAT-HOF@Co-HHTP model with interfacial Co-N_1_ bond is thus used for the subsequent calculations (Supplementary Data 1). The differential charge distribution diagram of the optimized heterostructure shows the electron accumulation around Co atom in Co-HHTP while electron depletion from N atom in DAF-HOF via the interfacial Co-N bridge (Fig. 5a), indicating the electron transfer from DAT-HOF to Co-HHTP, consistent with the XPS and UPS results.

To identify the 2e⁻ ORR active site in DAT-HOF@Co-HHTP, the O_2_ adsorption energies of different sites were calculated including Co^2+^, N_1_ and N_2_ (Fig. 5b). The free energy change (ΔG) of O_2_ adsorption on Co^2+^ was −0.51 eV, lower than that on N_1_ ( − 0.14 eV) and N_2_ (0.32 eV), suggesting that the Co^2+^ site is the active center for 2e⁻ ORR. Figure 5c, d depict the free-energy diagrams of 2e⁻ ORR and adsorption configurations of each step. The ΔG values of *O_2_ for DAT-HOF@Co-HHTP and Co-HHTP are −0.49 and −0.34 eV, respectively, suggesting the enhanced O_2_ adsorption and activation ability of DAT-HOF@Co-HHTP, in accordance with the observations of in-situ ATR-IR. For the second step of *O_2_ to OOH*, a lower energy barrier is found for DAT-HOF@Co-HHTP ( − 0.25 eV) than Co-HHTP ( − 0.19 eV, Supplementary Data 2), indicating that the O_2_ protonation over DAT-HOF@Co-HHTP is thermodynamically more favorable (Fig. 5c and Supplementary Fig. 18). For the second hydrogenation step from *OOH to *HOOH, the energy cost for DAT-HOF@Co-HHTP (0.22 eV) is also lower than that of Co-HHTP (0.29 eV), further underscoring the crucial role of DAT-HOF in facilitating proton supply. Moreover, the ΔG of *O formation via competitive 4e⁻ ORR pathway was calculated to be −0.51 eV for DAT-HOF@Co-HHTP, higher than that of H_2_O_2_ ( − 0.66 eV), indicating that its high 2e⁻ ORR selectivity has a thermodynamical origin. In contrast, the lower ΔG of O_ad_ than H_2_O_2_ results in the low 2e⁻ ORR selectivity for Co-HHTP, consistent with the electrochemical results.

Collectively, the construction of DAT-HOF@Co-HHTP heterostructured electrocatalyst significantly improves the 2e⁻ ORR performance for H_2_O_2_ production. Within the heterostructure, DAT-HOF with a protonated structure and abundant H-bond networks serves as an efficient proton pump while Co-HHTP improves electron conductivity. Upon the contact of two components, the difference in Fermi levels drives the spontaneous electron transfer from DAT-HOF to Co-HHTP through the interfacial Co−N bonds. The produced electron-sufficient Co active sites with optimized binding strength of oxygen intermediates play a crucial role in enhancing the 2e⁻ ORR activity and selectivity. Simultaneously, the charge redistribution results in the formation of an interfacial built-in electric field pointing from DAT-HOF to Co-HHTP, inducing the directional migration of protons from DAT-HOF to Co sites in Co-HHTP for O_2_ hydrogenation to H_2_O_2_. By integrating the finely tuned active sites, high electron conductivity and sufficient proton supply capability, DAT-HOF@Co-HHTP is capable of driving ampere-level 2e⁻ ORR process with high performance.

## Discussion

In this work, a DAT-HOF@Co-HHTP heterostructured electrocatalyst has been synthesized for H_2_O_2_ electrosynthesis with a high H_2_O_2_ production rate of 738.9 mg h⁻^1^ cm⁻^2^ and a H_2_O_2_ FE of 97.1 ± 0.4% at a current density of 1200 mA cm⁻^2^. The combination of Co-HHTP with DAT-HOF endows the heterostructure with high conductivity, sufficient proton supply and tailored active sites, synergistically promoting the 2e⁻ ORR to H_2_O_2_ production. Despite of the innovative HOF@*c*MOF heterostructure design, our work has demonstrated the importance of sufficient proton supply towards ampere-level electrocatalytic production of H_2_O_2_ via the 2e^–^ ORR route. To realize practical industrial H_2_O_2_ electrosynthesis, further work should be conducted such as scalable synthesis of the developed electrocatalysts, developing optimized reactor systems, establishing effective product extraction strategies, and conducting a comprehensive techno-economic analysis to assess scalability and cost-competitiveness.

## Methods

### Reagents and materials

N, N-dimethylformamide (DMF, AR, 99%, Greagent), 1H-1,2,4-triazole-3,5-diamine (DAT, >98.0%, Adamas-beta), naphthalene-1,4,5,8-tetracarboxylic dianhydride (NTD, >97.0%, Adamas-beta), N,N-dimethylacetamide (DMA, 99%, Greagent), cobalt(II) acetate tetrahydrate (Co(OAc)_2_·4H_2_O, 99%, Adamas-beta), 2,3,6,7,10,11-hexahydroxytriphenylene (HHTP, 99%, Adamas-beta), 1-propanol (C_3_H_8_O, ≥95.0%, Greagent) and ethanol (C_2_H_5_OH, 99%, Adamas-beta) were used as received. Millipore water was used in all experiments.

### Synthesis of DAT-HOF

DAT-HOF was synthesized according to the reported method^59^. Typically, 99.1 mg of DAT was dissolved into 10 mL of DMA by stirring in an ice bath under N_2_ atmosphere for 30 min. Afterwards, 134 mg of NTD and 60 mL of DMA were added into the above mixture with stirring for another 30 min. The suspension was then transferred into a 100 mL Teflon-line autoclave and heated at 170 °C for 6 h. Finally, the resultant DAT-HOF was collected by centrifugation, washed by DMA and ethanol for three times and dried at 60 °C for further use.

### Synthesis of DAT-HOF@Co-HHTP

To synthesize DAT-HOF@Co-HHTP, 8 mg of DAT-HOF and 7 mg of HHTP was ultrasonically dispersed into a mixture solution containing 1 mL of DMA, 4 mL of 1-propanol and 4 mL of H_2_O. Then, another mixture solution prepared by dissolving 6 mg of Co(OAc)_2_·4H_2_O in 4 mL of H_2_O and 4 mL of 1-propanol was poured into the above suspension. After heating at 55 °C for 1 h, the DAT-HOF@Co-HHTP powder was collected by centrifugation, washed with H_2_O and ethanol for three times, and dried at 60 °C.

### Synthesis of Co-HHTP

Co-HHTP was prepared by the similar process of DAT-HOF@Co-HHTP without the addition of DAT-HOF.

### Material characterization

Transmission electron microscopy (TEM) images were acquired by Hitachi HT7700 at 120 KV. Chemical composition analyses were carried out using JEM-2100F (JEOL, Japan) operating at 200 kV equipped with an X-ray energy dispersive spectrometer (EDS, X-Max 80 T, Oxford, UK). Field-emission scanning electron microscope (SEM) images were collected by scanning electron microscope (HITACHI-S4800). X-ray diffraction (XRD) patterns were recorded by a Bruker D8 Advanced X-Ray Diffractometer with Cu Kα radiation (λ = 0.154 nm). Fourier transform infrared (FTIR) spectra were collected on a Nicolet Fourier spectrophotometer using KBr pellets. X-ray photoelectron spectroscopy (XPS) studies were carried out on an AXIS Supra+ using an Al Kαradiation and C 1 s (284.8 eV) as a reference to correct the binding energy. The concentrations of metal ions were determined by Agilent 730 inductively coupled plasma-optical emission spectrometry (ICP-OES). UV-vis spectra were obtained by using a UV-vis spectrophotometer (Perkin Elmer Lambda 750). Ultraviolet photoelectron spectroscopy (UPS) studies were conducted by using an ESCALAB 250 XI analyzer with He (21.22 eV) as monochromatic light source. The X-ray absorption fine structure (XAFS) measurements were performed using the Rapid XAFS 1 M (Anhui Absorption Spectroscopy Analysis Instrument Co., Ltd.). Data analysis was performed with the Athena and Artemis programs of the Demeter data analysis packages that utilizes the FEFF6 program to fit the EXAFS data^60–62^.

### Electrochemical measurement

#### The ORR activities evaluated in rotating ring-disk electrode (RRDE) system

Rotating ring-disk electrode (RRDE) tests were performed using a standard three-electrode system on a CHI-760C electrochemical workstation (CH Instruments Inc.) in O_2_-saturated 0.1 M K_2_SO_4_ solution (200 mL, pH=7 ± 0.2) at ambient temperature (25 ± 1 °C). The electrolyte was freshly prepared for each measurement. Platinum wire (CH, CHI115), Ag/AgCl (KCl, 3.5 M, CHI111) and catalyst-modified glassy carbon were applied as the counter, reference and working electrodes, respectively. The Ag/AgCl reference electrode is calibrated using Potassium Ferrocyanide/Ferricyanide (K_4_[Fe(CN)_6_]/K_3_[Fe(CN)_6_]) couple. The catalyst inks were prepared by dispersing 10 mg of sample into 1 mL of isopropanol containing 40 μL of Nafion solution to form a homogeneous suspension. The obtained ink was then dipped onto the polished glassy carbon disk (0.2475 cm⁻^2^) under an infrared lamp. The loading amount of catalyst is determined to be 0.2 mg cm^−2^. The RRDE was rotated at 1600 rpm throughout the whole tests. Liner sweep voltammetry (LSV) curves were recorded at a scan rate of 5 mV s^−1^. A constant voltage of 1.2 V vs. RHE was applied to the ring electrode. The potential reaching the current density of 0.1 mA cm^−2^ in polarization curves on disk electrode was defined as the onset potential. The selectivity of H_2_O_2_ was calculated using the following equation: Selectivity (%) = 200 × (I_r_/N)/(I_d_+I_r_/N). The electron transfer number (n) is calculated by the following equation:

1 $${{\rm{n}}}=4\times \frac{{{{\rm{I}}}}_{{{\rm{d}}}}}{{{{\rm{I}}}}_{{{\rm{d}}}}+{{{\rm{I}}}}_{{{\rm{d}}}}/{{\rm{N}}}}$$

where I_r_ is the ring current, I_d_ is the disk current and N is the current collection efficiency of the Pt ring electrode (N = 0.256).

The Tafel slope (b) was obtained by fitting the linear part of the Tafel plots according to the Tafel equation

2 $${{\rm{\eta }}}={{\rm{a}}}+{{\rm{blog}}}({{\rm{j}}})$$

to evaluate the kinetic performance of as-prepared catalysts for ORR. The electrochemical active surface area (ECSA) was evaluated based on the double-layer capacitances (C_dl_) of the catalysts on RDE by cyclic voltammograms (CV) curves at different scanning rates of 10–100 mV s^−1^ in the non-Faradaic voltage region. A straight line can be obtained by plotting the current density against the scan rate at a specific potential in the CV curves. The slope of the line is defined as electrochemical double-layer capacitance (C_dl_). Furthermore, the ECSA can be calculated as:

3 $${{\rm{ECSA}}}=\frac{{{{\rm{C}}}}_{{{\rm{dl}}}}}{{{\rm{A}}}\times {{\rm{Cs}}}}$$

where A is the amount of the material coating on the surface of electrode (mg cm^−2^), Cs is an empirical constant representing the capacitance per unit area (40 μF cm^−2^). Electrochemical impedance spectroscopy (EIS) was measured in 0.1 M K_2_SO_4_ solution in the frequency range of 1000 kHz to 0.01 Hz with an amplitude of 10 mV. All the potentials were calibrated with a reversible hydrogen electrode (RHE)

4 $${{{\rm{E}}}}_{{{\rm{RHE}}}}={{{\rm{E}}}}_{{{\rm{Ag}}}/{{\rm{AgCl}}}}+0.0591\times {{\rm{pH}}}+0.197$$

All the electrochemical tests were performed without IR compensation.

### Electrochemical evaluation in the flow-type cell

Electrochemical tests were carried out on a CHI760E electrochemical workstation connected to a CHI680D high current amplifier at ambient temperature (25 ± 1 °C). A standard three-electrode three-phase flow cell system was assembled by employing gas diffusion electrode (GDE) as a working electrode, platinum electrode as a counter electrode, and Ag/AgCl (KCl, 3.5 M, CHI111) as a reference electrode. The catalyst inks were prepared by dispersing 10 mg of sample into 1 mL of isopropanol containing 40 μL of Nafion solution to form a homogeneous suspension. Next, the homogeneous catalyst ink was dripped on GDE (chuxi) with an overall area of 2 × 2 cm^2^ (active area of 1 × 1 cm^2^). The loading amount of catalyst is determined to be 0.2 mg cm^−2^. The catholyte and anolyte were both 0.1 M K_2_SO_4_ aqueous solution (500 mL, pH=7 ± 0.2). The electrolyte was freshly prepared for each measurement. The electrolytic cell is separated by an anion exchange membrane (FunasepFAA-3-PK-130, 130 μm, 1 × 1 cm^2^). The membrane is pretreated by immersion in a 0.5 M NaCl solution at 25 °C for 24 h. A peristaltic pump was used to circulate the electrolyte with a rotational speed of 30 rpm. The O_2_ gas flow was maintained as 20 mL min^−1^ during the whole measurement. The H_2_O_2_ production rate was determined by the iodometry method. Typically, 100 μL of reaction solution was collected from the electrochemical system and subsequently added to the mixture of potassium hydrogen phthalate (C_8_H_5_KO_4_) and potassium iodide (KI) aqueous solution with reaction for 30 min. The H_2_O_2_ were allowed to react with I^−^ to generate I_3_^−^ (H_2_O_2_ + 3I^− ^+ 2H^+^ → I_3_^− ^+ H_2_O). The amount of I_3_^−^ was measured by a Synergy-H1 microplate reader at its characteristic absorbance peak of 350 nm for H_2_O_2_ quantification. All the electrochemical tests were performed without IR compensation.

### Computational details

The Density Functional Theory (DFT) calculations were conducted using the Vienna Ab-inito Simulation Package (VASP)^63,64^. The Perdew-Burke-Ernzerhof (PBE) with the generalized gradient approximation (GGA) method was employed to describe the exchange-correlation effects^65,66^. The core-valence interactions were calculated by the projected augmented wave (PAW) method^67^. An energy cutoff of 500 eV was set for plane wave expansions, and the 3 × 2 × 1 Monkhorst-Pack grid k-points were selected to sample the Brillouin zone integration. The vacuum space is adopted 15 Å above the surfaces to avoid periodic interactions. The structural optimization was completed for energy and force convergence set at 1.0 × 10^−4^ eV and 0.02 eV Å^−1^, respectively. The models were constructed based on the standard crystalline structure of DAT-HOF and Co-HHTP.

The adsorption energy can be calculated according to the following formula:

5 $${{\rm{Eads}}}={{\rm{E}}}({{\rm{A}}}+{{\rm{B}}})-{{\rm{E}}}({{\rm{A}}})-{{\rm{E}}}({{\rm{B}}})$$

where Eads represents the adsorption energy, E(A + B) is the calculated energy of adsorption configuration, E(A) and E(B) mean the calculated energy of substrate and adsorbent respectively.

The Gibbs free energy change (ΔG) of each step is calculated using the following formula:

6 $$\Delta {{\rm{G}}}=\Delta {{\rm{E}}}+\Delta {{\rm{ZPE}}}-{{\rm{T}}}\Delta {{\rm{S}}}$$

where ΔE is the electronic energy difference directly obtained from DFT calculations, ΔZPE is the zero point energy difference, T is the room temperature (298.15 K) and ΔS is the entropy change. ZPE could be obtained after frequency calculation by ref. ^68^:

7 $${{\rm{ZPE}}}=\frac{1}{2}\sum {hvi}$$

The TS values of adsorbed species are calculated according to the vibrational frequencies^69^:

8 $${{\rm{TS}}}={k}_{B}T\left[{\sum }_{k}{{\rm{In}}}\left(\frac{1}{1-{e}^{-{hv}/{k}_{B}T}}\right)+{\sum }_{k}\frac{{hv}}{{k}_{B}T}\frac{1}{({e}^{{hv}/{k}_{B}T}-1)}+1\right]$$

## Supplementary information

Supplementary Information

Description of Additional Supplementary Files

Supplementary Data 1

Supplementary Data 2

Transparent Peer Review file

## Source data

Source Data

## Data Availability

The raw data generated in this study are provided in the Supplementary Information. All data are available from the corresponding author upon request. Source data are provided with this paper Source data are provided with this paper.
