# Supplementary material for: Hydrogen-bonded organic framework@conductive metal-organic framework heterostructures for ampere-level hydrogen peroxide production
Source: Nat Commun. 2025 Dec 4;16:10908. doi: 10.1038/s41467-025-65887-6 (PMC12678433; doi:10.1038/s41467-025-65887-6)
Supplement: Supplementary file 1 — Supplementary Information [file 41467_2025_65887_MOESM1_ESM.pdf]

Supplementary information

Hydrogen-Bonded Organic Framework@Conductive Metal-Organic Framework Heterostructures for Ampere-Level Hydrogen Peroxide Production

Yingying Zou[1], Yulin Zhang[1], Chaoqi Zhang[1], Tong Bao[1], Yamin Xi[1], Niqi Ao[1], Zhijie Li[1], Yunying Wang[1], Chao Liu[1,2,3]*, Chengzhong Yu[1,2,4]*

[1] School of Chemistry and Molecular Engineering, East China Normal University; Shanghai 200241, P. R. China.

[2] State Key Laboratory of Petroleum Molecular and Process Engineering, SKLPMPE, East China Normal University, Shanghai 200062, P. R. China.

[3] Shanghai Frontiers Science Center of Molecule Intelligent Syntheses, School of Chemistry and Molecular Engineering, East China Normal University, Shanghai, 200062, China.

[4] Australian Institute for Bioengineering and Nanotechnology, The University of Queensland; Brisbane, Queensland 4072, Australia.

*Correspondence: cliu@chem.ecnu.edu.cn; c.yu@uq.edu.au

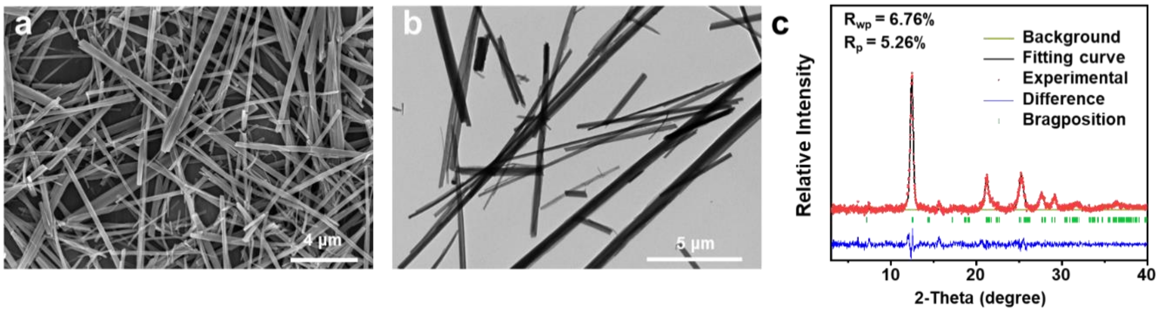

Supplementary Figure 1. a SEM and **b** TEM images, and **c** XRD pattern of DAT-HOF. Source data for Supplementary Figure 1 are provided as a Source Data file.

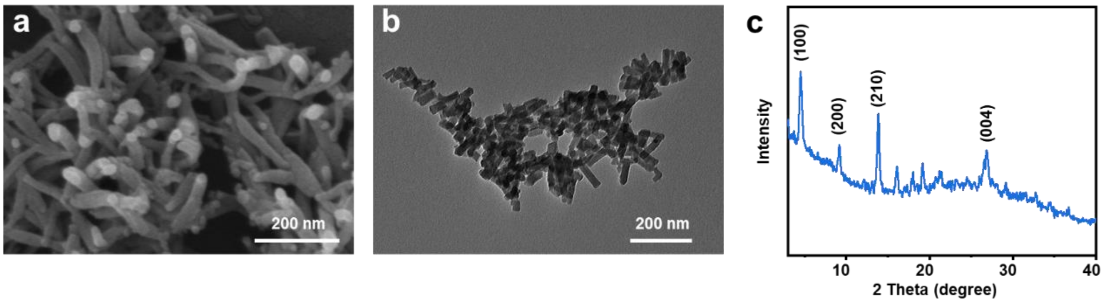

Supplementary Figure 2. a SEM and **b** TEM images, and **c** XRD pattern of Co-HHTP. Source data for Supplementary Figure 2 are provided as a Source Data file.

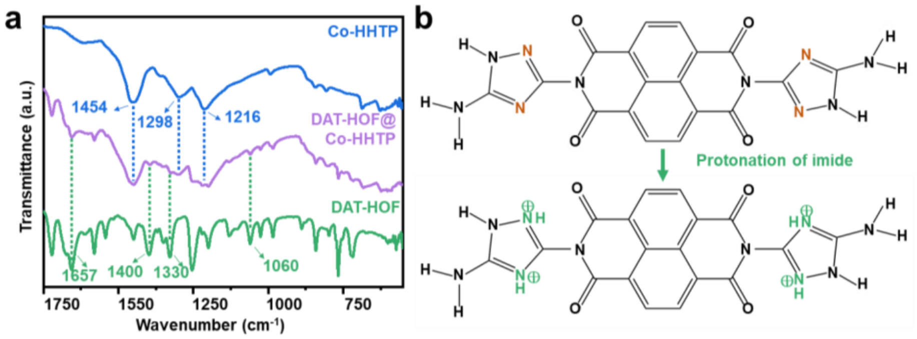

Supplementary Figure 3. a FTIR spectra of DAT-HOF@Co-HHTP, DAT-HOF and Co-HHTP. **b** Structure of DAT-HOF with protonated C=N groups. Source data for Supplementary Figure 3 are provided as a Source Data file.

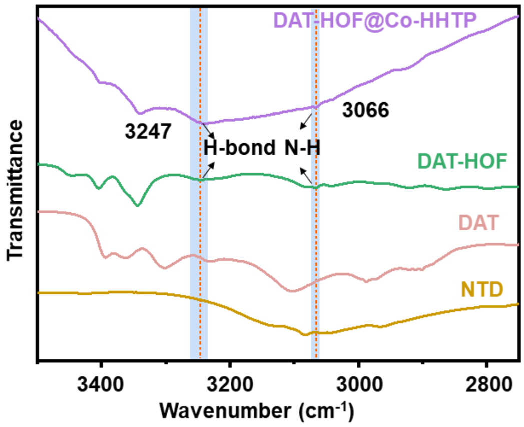

Supplementary Figure 4. FTIR spectra of DAT-HOF@Co-HHTP, DAT-HOF and Co-

HHTP. Source data for Supplementary Figure 4 are provided as a Source Data file.

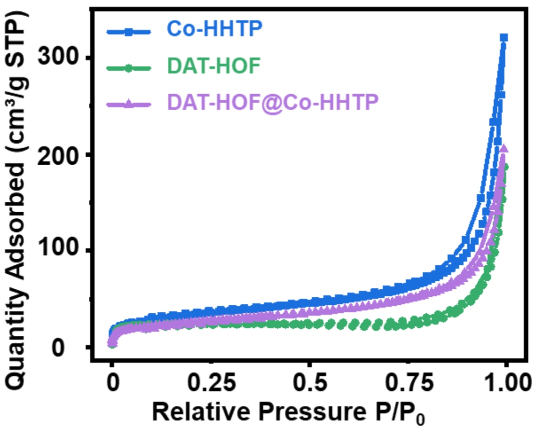

Supplementary Fig. 5. Nitrogen adsorption-desorption isotherms of DAT-HOF, Co-HHTP and DAT-HOF@Co-HHTP. Source data for Supplementary Figure 5 are provided as a Source Data file.

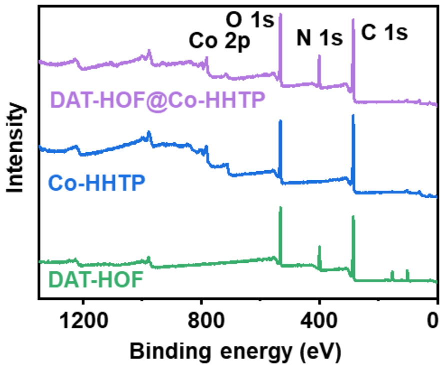

Supplementary Figure 6. XPS survey spectra of DAT-HOF@Co-HHTP, DAT-HOF and Co-HHTP. Source data for Supplementary Figure 6 are provided as a Source Data file.

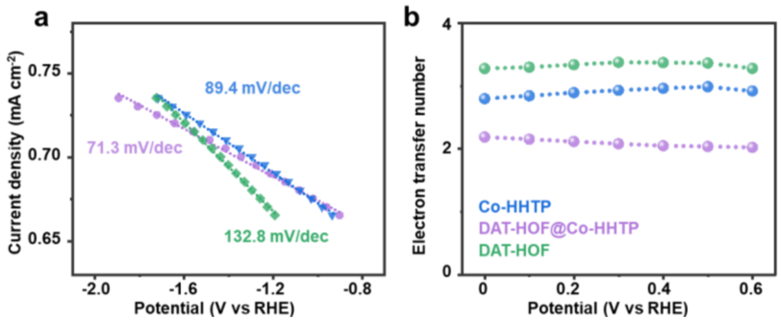

Supplementary Figure 7. a Tafel slope curves and **b** electron transfer numbers of different samples. Source data for Supplementary Figure 7 are provided as a Source Data file.

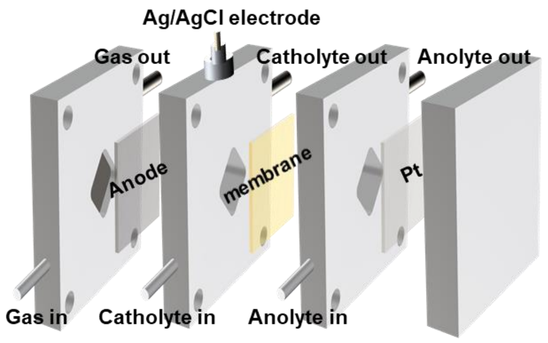

Supplementary Figure 8. Scheme of electrochemical flow-type cell.

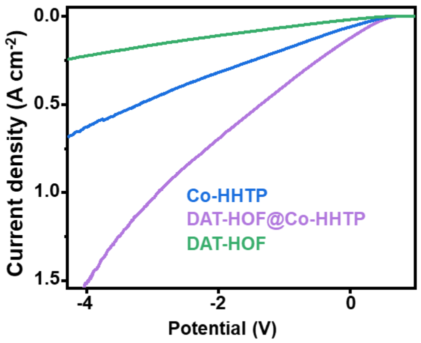

Supplementary Figure 9. LSV polarization curves of DAT-HOF@Co-HHTP, DAT-HOF and Co-HHTP in flow cell. Source data for Supplementary Figure 9 are provided as a Source Data file.

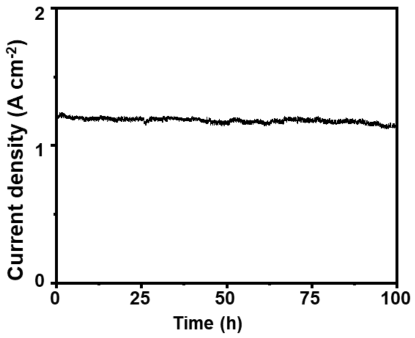

Supplementary Figure 10. Durability test under -3.9 V vs RHE for DAT-HOF@Co-HHTP. Source data for Supplementary Figure 10 are provided as a Source Data file.

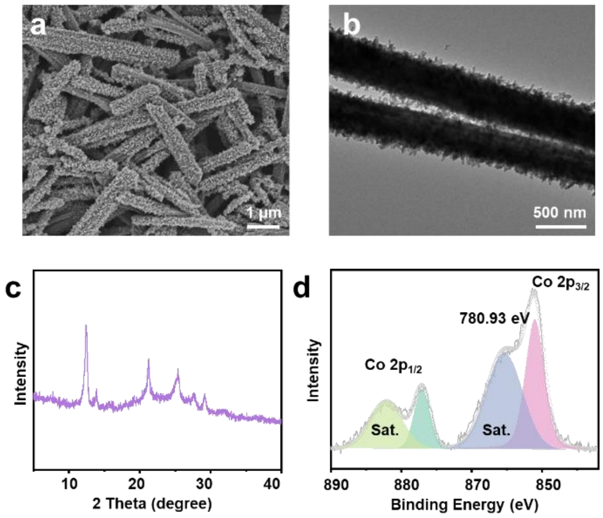

Supplementary Figure 11. **a** SEM and **b** TEM images, **c** XRD pattern and **d** XPS spectrum of DAT-HOF@Co-HHTP after stability test. Source data for Supplementary Figure 11 are provided as a Source Data file.

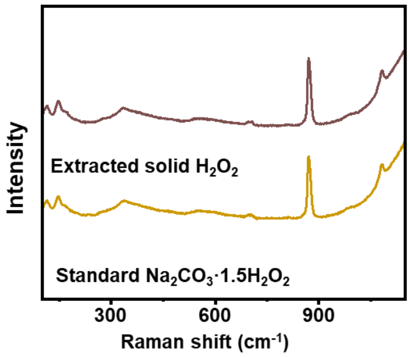

Supplementary Figure 12. Raman spectra of extracted solid H_2O_2 and standard $Na_2CO_3{\cdot}1.5H_2O_2$. Source data for Supplementary Figure 12 are provided as a Source Data file.

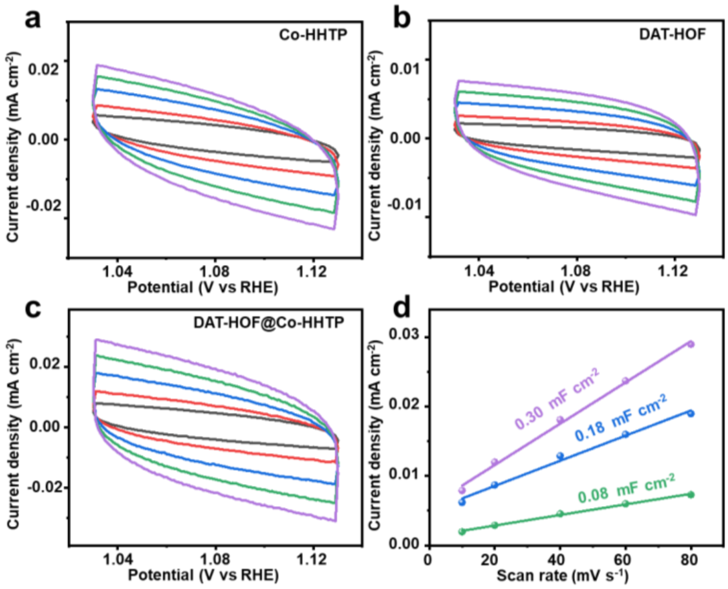

Supplementary Figure 13. CV curves of **a** Co-HHTP, **b** DAT-HOF and **c** DAT-HOF@Co-HHTP-3 at different scan rates. **d** CV current density versus scan rate of different samples, the liner slope is equivalent to the double-layer capacitance (C_{dl}). Source data for Supplementary Figure 13 are provided as a Source Data file.

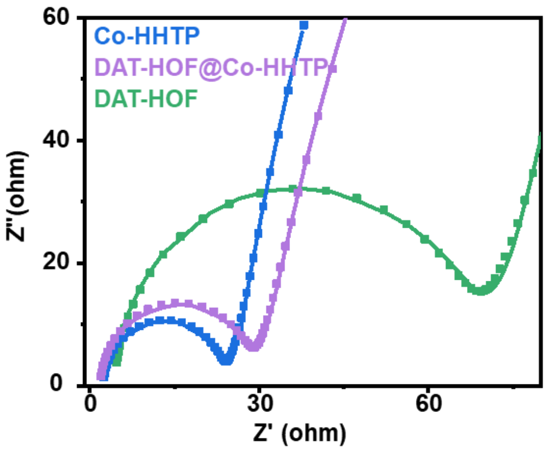

Supplementary Figure 14. EIS spectra of DAT-HOF, Co-HHTP and DAT-HOF@Co-HHTP. Source data for Supplementary Figure 14 are provided as a Source Data file.

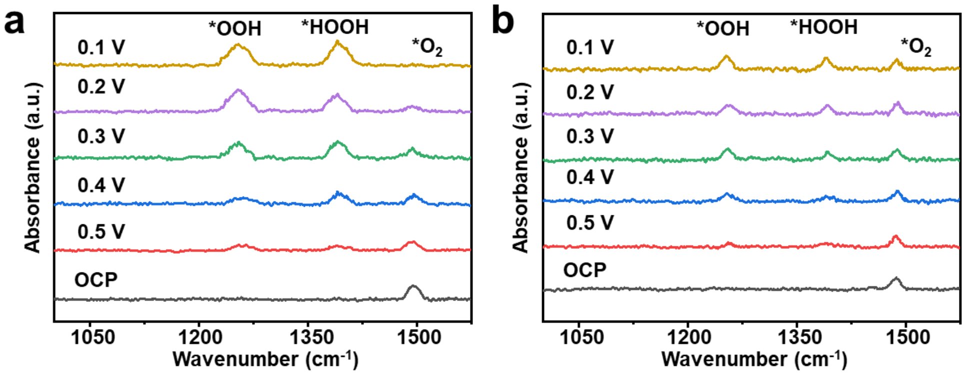

Supplementary Figure 15. In situ ATR-IR spectra of **a** DAT-HOF@Co-HHTP and **b** Co-HHTP at different applied potentials. Source data for Supplementary Figure 15 are provided as a Source Data file.

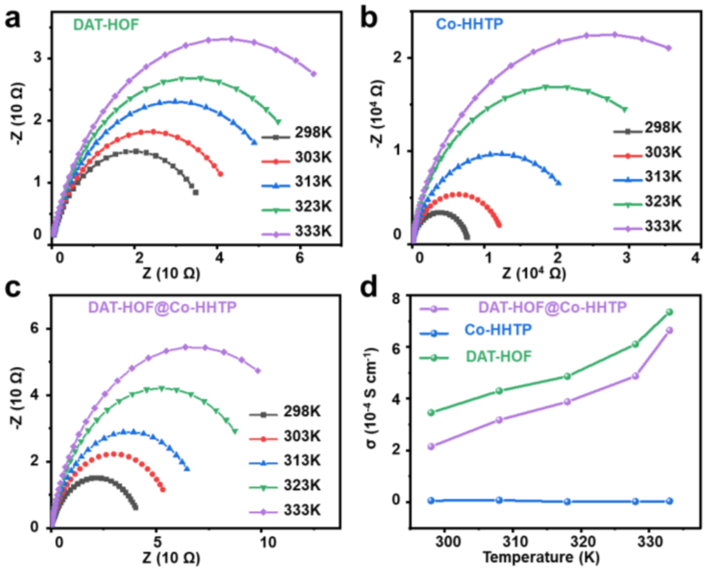

Supplementary Figure 16. Temperature-dependent Nyquist plots of **a** DAT-HOF, **b** Co-HHTP and **c** DAT-HOF@Co-HHTP at 293-333 K under 95 % relative humidity. **d** Proton conductivity of DAT-HOF, Co-HHTP and DAT-HOF@Co-HHTP. Source data for Supplementary Figure 16 are provided as a Source Data file.

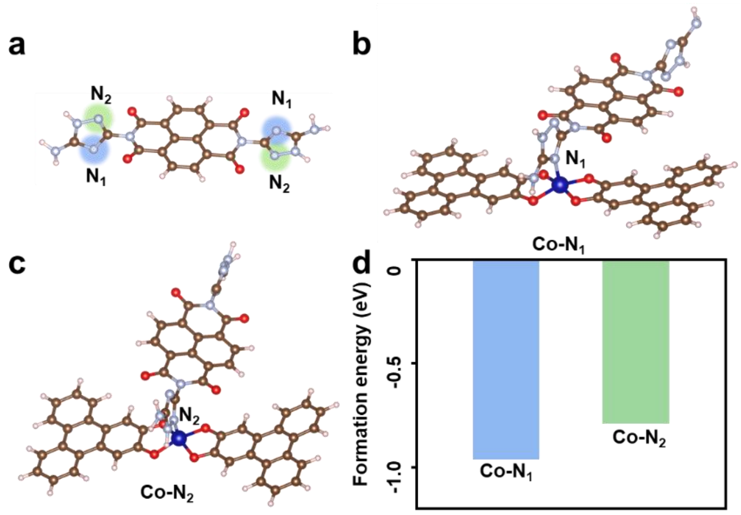

Supplementary Figure 17. a Two types of imide N atoms in DAT-HOF. Calculated models of DAT-HOF@Co-HHTP heterostructure with different binding sites: **b** Co-N_1 and **c** Co-N_2, and **d** the corresponding formation energies. Source data for Supplementary Figure 17 are provided as a Source Data file.

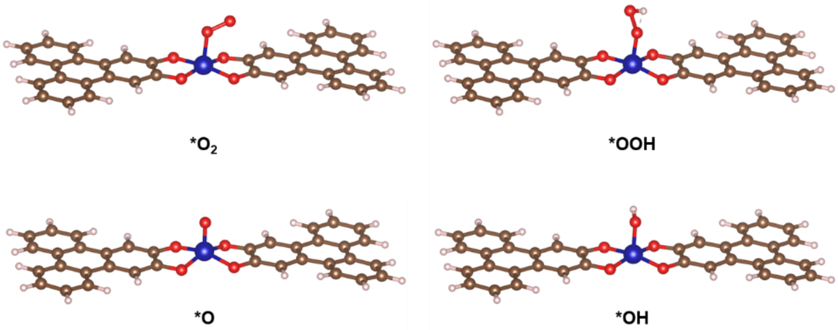

Supplementary Figure 18. Adsorption diagrams of the 2e⁻ ORR process over Co-HHTP.

Supplementary Table 1. Performance comparison of DAT-HOF@Co-HHTP with reported 2e⁻ ORR electrocatalysts.

**Catalyst**	**Current density ($A\ cm^{-2}$)**	**Productivity ($mg\ h^{-1}\ cm^{-2}$)**	**FE (%)**	**Electrolyte**	**Ref**
DAT-HOF@Co-HHTP	1.2	738.9	97.1	0.1 M K_2SO_4	This work
F-Cu-MOF	2	1082	84.9	0.6 M K_2SO_4	1
L-ZnO	1	624.15	98.48	0.6 M K_2SO_4	2

ZnO-v	1	621.88	98.1	0.6 M K_2SO_4	3
TiO_xF_y	1	614	96.4	0.6 M K^+ (pH=13)	4
CoPc/CNT	0.2	112.2	88.7	0.1 M KPi	5
C-0.1M80	0.2	123.7	97.5	0.1 M KOH	6
VG array	0.1	61.3	94	0.1 M KOH	7
BP2000	0.4	211.14	83	0.01 M Na_2SO_4 + 0.1 M H_2SO4	8
PTFE/CB NADEs	0.06	30.94	81.3	0.05 M Na_2SO4	9
ZnCo-ZIF-C3	0.07	78.812	85	0.1 M PBS	10
Sb-NSCF	0.05	25.364	94.7	0.1 M KOH	11
SNC	0.15	13.265	70	0.1 M $HClO_4$	12
Pd/MCS-8	0.245	137.84	88.7	0.5 M K_2SO4	13
E-BPC	0.3	202.55	85.14	1 M Na_2SO4	14
Ir-Ta-Ti	0.11	64.08	91.8	0.1 M KOH	15
$Mg_3(HITP)_2$	0.1	142.8	85	0.1 M PBS	16

Reference

1. Li, Q.; Nie, Z.; Wu, W.; Guan, H.; Xia, B.; Huang, Q.; Duan, J.; Chen, S. Water Spillover to Expedite Two-Electron Oxygen Reduction. *Adv. Mater.* **37**, 2412039 (2025).

2. S. Ding, B. Xia, M. Li, F. Lou, C. Cheng, T. Gao, Y. Zhang, K. Yang, L. Jiang, Z. Nie, H. Guan, J. Duan, S. Chen. *Energy Environ. Sci.* **16**, 3363 (2023).

3. S. Ding, Y. Zhang, F. Lou, M. Li, Q. Huang, K. Yang, B. Xia, C. Tang, J. Duan, M. Antonietti, S. Chen. *Mater. Today Energy* **38**, 101430 (2023).

4. B. Xia, J. Du, M. Li, J. Duan, S. Chen. *Adv. Mater.* **36**, 2401641 (2024).

5. Y. Lee, C. Lee, S. Back, Y. J. Sa. *Nanoscale* **16**, 9545 (2024).

6. S. Jia, H. Yu, J. Na, Z. Liu, K. Lv, Z. Ren, S. Sun, Z. Shao. *ACS Appl. Mater. Interfaces* **16**, 23099 (2024).

7. Y. Wang, R. Shi, L. Shang, L. Peng, D. Chu, Z. Han, G. I. N. Waterhouse, R. Zhang, T. Zhang. *Nano Energy* **96**, 107046 (2022).

8. X. Zhang, X. Zhao, P. Zhu, Z. Adler, Z.-Y. Wu, Y. Liu, H. Wang. *Nat. Commun.* **13**, 2880 (2022).

9. Q. Zhang, M. Zhou, G. Ren, Y. Li, Y. Li, X. Du. *Nat. Commun.* **11**, 1731 (2020).

10. C. Zhang, L. Yuan, C. Liu, Z. Li, Y. Zou, X. Zhang, Y. Zhang, Z. Zhang, G. Wei, C. Yu. *J. Am. Chem. Soc.* **145**, 7791 (2023).

11. M. Yan, Z. Wei, Z. Gong, B. Johannessen, G. Ye, G. He, J. Liu, S. Zhao, C. Cui, H. Fei. *Nat. Commun.* **14**, 368 (2023).

12. Z. Mou, Y. Mu, L. Liu, D. Cao, S. Chen, W. Yan, H. Zhou, T. S. Chan, L. Y. Chang, X. Fan. *Small* **20**, 2400564 (2024).

13. L. Y. Jing, W. Y. Wang, Q. Tian, Y. Kong, X. S. Ye, H. P. Yang, Q. Hu, C. X. He. *Angew. Chem., Int. Ed.* **63**, e202403023 (2024).

14. A. Byeon, J. W. Choi, H. W. Lee, W. C. Yun, W. Zhang, C.-K. Hwang, S. Y. Lee, S. S. Han, J. M. Kim, J. W. Lee. *Appl. Catal., B* **329**, 122557 (2023).

15. C. Jiang, Y.-F. Fei, W. Xu, Z. Bao, Y. Shao, S. Zhang, Z.-T. Hu, J. Wang. *Appl. Catal., B* **334**, 122867 (2023).

16. K. Dong, J. Liang, Y. Wang, L. C. Zhang, Z. Q. Xu, S. J. Sun, Y. S. Luo, T. S. Li, Q. Liu, N. Li, B. Tang, A. A. Alshehri, Q. Li, D. W. Ma and X. P. Sun. Conductive two-dimensional magnesium metal-organic frameworks for high-efficiency O_2 electroreduction to H_2O_2, *ACS Catal.* **12**, 6092-6099 (2022).
