# Supplementary material for: Hydrogen-bonded organic framework@conductive metal-organic framework heterostructures for ampere-level hydrogen peroxide production
Source: Nat Commun. 2025 Dec 4;16:10908. doi: 10.1038/s41467-025-65887-6 (PMC12678433; doi:10.1038/s41467-025-65887-6)
Supplement: Supplementary file 2 — Description of Additional Supplementary Files [file 41467_2025_65887_MOESM2_ESM.pdf]

Description of Additional Supplementary Files

File Name: Supplementary Data 1

Description: CONTCAR-Co-HHTP.

Calculated model of Co-HHTP.

File Name: Supplementary Data 2

Description: CONTCAR-DAT-HOF-Co-HHTP.

Calculated model of DAT-HOF@Co-HHTP heterostructure.
